# Development and validation of an endoplasmic reticulum stress-related molecular prognostic model for breast cancer

**DOI:** 10.3389/fonc.2023.1178595

**Published:** 2023-05-29

**Authors:** Pengyu Fan, Jiajia Wang, Ruolei Li, Kexin Chang, Liuyin Liu, Yaping Wang, Zhe Wang, Bo Zhang, Cheng Ji, Jian Zhang, Suning Chen, Rui Ling

**Affiliations:** ^1^Department of Thyroid, Breast and Vascular Surgery, Xijing Hospital, Fourth Military Medical University, Xi’an, China; ^2^The State Key Laboratory of Cancer Biology, Department of Biochemistry and Molecular Biology, The Fourth Military Medical University, Xi’an, China; ^3^Department of Pharmacy, Xijing Hospital, The Fourth Military Medical University, Xi’an, Shaanxi, China

**Keywords:** bioinformatic analysis, breast cancer, endoplasmic reticulum stress, gene signature, immune infiltrate cells, overall survival

## Abstract

**Background:**

Breast cancer is the most frequently diagnosed cancer and a leading cause of cancer-related death in women. Endoplasmic reticulum stress (ERS) plays a crucial role in the pathogenesis of several malignancies. However, the prognostic value of ERS-related genes in breast cancer has not been thoroughly investigated.

**Methods:**

We downloaded and analyzed expression profiling data for breast invasive carcinoma samples in The Cancer Genome Atlas-Breast Invasive Carcinoma (TCGA-BRCA) and identified 23 ERS-related genes differentially expressed between the normal breast tissue and primary breast tumor tissues. We constructed and validated risk models using external test datasets. We assessed the differences in sensitivity to common antitumor drugs between high- and low-scoring groups using the Genomics of Drug Sensitivity in Cancer (GDSC) database, evaluated the sensitivity of patients in high- and low-scoring groups to immunotherapy using the Tumor Immune Dysfunction and Exclusion (TIDE) algorithm, and assessed immune and stromal cell infiltration in the tumor microenvironment (TME) using the Estimation of Stromal and Immune cells in Malignant Tumor tissues using Expression data (ESTIMATE) algorithm. We also analyzed the expression of independent factors in the prognostic model using the Western-blot analysis for correlation in relation to breast cancer.

**Results:**

Using multivariate Cox analysis, *FBXO6*, *PMAIP1*, *ERP27*, and *CHAC1* were identified as independent prognostic factors in patients with breast cancer. The risk score in our model was defined as the endoplasmic reticulum score (ERScore). ERScore had high predictive power for overall survival in patients with breast cancer. The high-ERScore group exhibited a worse prognosis, lower drug sensitivity, and lower immunotherapy response and immune infiltration than did the low-ERScore group. Conclusions based on ERScore were consistent with Western-blot results.

**Conclusion:**

We constructed and validated for the first time an endoplasmic reticulum stress-related molecular prognostic model for breast cancer with reliable predictive properties and good sensitivity, as an important addition to the prognostic prediction model for breast cancer.

## 1 Introduction

Global cancer statistics for 2020 revealed that breast cancer is the most prevalent form of cancer, with approximately 2.26 million new cases annually, accounting for 11.7% of total cancer diagnoses. Breast cancer is the most commonly diagnosed cancer in women and one of the leading causes of cancer-related mortality (1). Conventional treatment for breast cancer involves surgery combined with chemotherapy, radiotherapy, endocrine drug therapy, molecular-targeted drug therapy, or immunotherapy, depending on the clinical tumor subtypes. Individualized treatment is also provided to patients in particular cases (2). Despite advances, this disease remains the leading cause of death in women, with approximately 3–10% of patients newly diagnosed with breast cancer showing distant metastases at the time of diagnosis. Furthermore, approximately 30% of patients diagnosed at early-stage progress to advanced breast cancer. The five-year survival rate for patients diagnosed at a late stage is only 20%, and the overall median survival time is 2–3 years (3). Therefore, further comprehensive and objective criteria are required to evaluate molecular mechanisms underlying the development of breast cancer, identify prognostic indicators, and discover novel diagnostic and therapeutic prognostic targets and assessments for patients with breast cancer.

The endoplasmic reticulum(ER), one of the largest organelles in eukaryotic cells, is a network of branching tubules and flattened vesicles interconnected by a closed space called the ER lumen, which is separated from the surrounding cytoplasm by a lipid bilayer ER membrane (4). The ER is involved in dynamic cellular functions, controlling lipid metabolism, calcium storage, and protein homeostasis. The ER plays a major role in the synthesis, folding, and structural maturation of at least one-third of the proteins in the cell (5). Widespread cellular stress affects the efficiency of protein folding in the ER. It leads to the accumulation of misfolded proteins within this organelle, including nutrient deprivation, hypoxia, point mutations in secreted proteins that stabilize intermediate folded forms or cause aggregation, and loss of calcium homeostasis (6). This overburdening is a sign of ERS (7). ERS induces an adaptive response called the unfolded protein response. Three major stress sensors, inositol acquisition enzyme 1α (IRE1α), the protein kinase RNA-like ER kinase (PERK), and activating transcription factor 6 (ATF6), control the unfolded protein response (8). These transmembrane ER proteins transmit signals to the cell membrane and nucleus through various pathways to restore the folding capacity for proteins. ERS plays a crucial role in the pathogenesis of malignancies and is involved in the development and progression of solid tumors (9). ERS influences multidrug resistance, metastasis, immunotherapy, and apoptosis in breast cancer (10–12), suggesting that ERS plays a critical role in the progression of breast cancer. Although some preclinical studies have shown promising results in treating breast cancer, inhibiting metastasis, and reversing chemotherapy resistance by targeting ERS-related molecules (10, 11, 13), we lack a practical predictive model of BRCA-related ERS to assess treatment efficacy and prognosis in patients with breast cancer. In the treatment of patients with malignancies, improvement in overall survival(OS) and disease-free survival (DFS) is of considerable significance. Constructing individualized prognostic models for ERS-related genes based on clinical samples for patients with breast cancer is vital.

Accordingly, we first performed clustering analysis based on The Cancer Genome Atlas (TCGA)-BRCA cohort. Subsequently, we screened differentially expressed genes (DEGs) for ERS by comparing normal breast tissue and primary breast cancer samples. Subsequently, we identified independent prognostic factors and developed an ERS score (ERScore) model. We analyzed the relationship between ERScore and drug sensitivity, as well as immune infiltration, and developed a clinical prediction model based on ERScore. We believe that our findings will help predict patient prognosis and provide a reference for clinical chemotherapy and immunotherapy and may provide novel insights into survival prediction and treatment strategies for patients with breast cancer.

## 2 Materials and methods

### 2.1 Data collection

Expression profiling data comprising raw counts and fragments per kilobase of exon per million mapped fragments (FPKM) values for breast cancer samples in the TCGA-BRCA dataset was downloaded from the UCSC Xena database (http://xena.ucsc.edu/), and FPKM values were normalized to transcript per million (TPM) values. The TCGA-BRCA dataset contains transcriptomic data from 1,217 patients with breast cancer, of which 1,072 primary tumor (01A) and 99 normal (11A) samples were included in the analysis(**Table 1**). “Masked Somatic Mutation” data from the TCGA Genomic Data Commons website (https://portal.gdc.cancer.gov/) were taken as somatic mutation data for breast invasive carcinoma (n = 1,044). The data were pre-processed using VarScan(https://sourceforge.net/projects/varscan/files/) and visualized using the maftools R package (14). We downloaded “Masked Copy Number Segment” data (n = 1098) and analyzed the copy number variation of genes in TCGA-BRCA dataset using the TCGAbiolinks R package (15). The downloaded CNV fragment data were analyzed using GenePattern (https://cloud.genepattern.org) (16) and the GISTIC 2.0 module was used to identify significant differences between groups. Clinical information for patients represented in the TCGA-BRCA dataset, including age, survival status, follow-up time, and pathological staging and typing, was also downloaded from the UCSC Xena database. Information on mutation and clinical characteristics of the patients was matched, and clinical data from 1,050 patients with breast cancer were included for further analysis.

**Table 1:** Baseline information for patients represented in the TCGA-BRCA dataset.

Characteristic	Low-ERScore	High-ERScore
n	525	525
Survival status (overall survival), n (%)		
0	461 (43.9%)	441 (42%)
1	64 (6.1%)	84 (8%)
age, n (%)		
<=60	276 (26.3%)	312 (29.7%)
>60	249 (23.7%)	213 (20.3%)
stage, n (%)		
stage I	107 (10.4%)	69 (6.7%)
stage II	306 (29.8%)	288 (28%)
stage III	99 (9.6%)	139 (13.5%)
stage IV	6 (0.6%)	14 (1.4%)
Survival time (overall survival), median (IQR)	1000 (532, 1980)	715 (426, 1492)

Additionally, we retrieved and downloaded from the Gene Expression Omnibus(GEO) database the GSE88770 (17) and GSE20685 (18) breast cancer datasets containing survival data. GSE88770 and GSE20685 contained transcriptomic and survival data from 117 and 327 patients, respectively, which were included in the analysis. The license of these datasets is GPL570. Further details are presented in **Table S1**.

### 2.2 Screening of differentially-expressed ERS genes and determination of ERScore

Two collections of ERS-related genes, “GO RESPONSE TO ENDOPLASMIC RETICULUM STRESS” and “GO REGULATION OF RESPONSE TO ENDOPLASMIC RETICULUM STRESS,” were downloaded from the Molecular Signature Database v7.0 (MSigDB) (18). Overlapping genes were removed to obtain 272 ERS-related genes. We used the Wilcoxon test to access differences in ERS gene expression between normal (11A) and primary breast cancer samples (01A) in the TCGA-BRCA dataset. Statistical significance was set at p< 0.05.

We used the least absolute shrinkage and selection operator (LASSO) algorithm to eliminate multicollinearity in the analysis based on differentially expressed ERS-related genes. We then screened independent prognostic factors using multi-factor Cox regression stepwise (stepwise, method = “both”) and built an ERScore model. The scoring formula was as follows:


$$\text{risk}Score\;=\sum_{i}Coefficient\;\left(hub\;gene_{i}\right)*mRNA\;Expression\;\left(hub\;gene_{i}\right)$$

The risk score obtained is termed “ERScore”. We used time-dependent receiver operating characteristic (ROC) curves to assess the probability of survival and calculated the area under the curve (AUC) for the ROC curves using the timeROC R package (19). We validated the Cox model using timeROC and the survival curves using data from GSE20685 and GSE88770 to assess stability and reliability.

### 2.3 Expression characteristics and clinical relevance of ERS genes

We evaluated ERS-associated genes with independent prognostic features obtained using multivariate Cox analysis in BRCA and 32 other cancer types, as well as alterations in relation to clinical features at transcriptome and mutation levels. The Kruskal-Wallis test was used to compare multiple groups, and p<0.05 was considered statistically significant.

### 2.4 Analysis of DEGs and functional enrichment in the ERS model

Patients represented in the TCGA-BRCA dataset were divided into high- (n = 525) and low- (n = 525) ERScore groups based on the median ERScore value, and differences in samples from the two groups were analyzed using the DESeq2 R package (20). An absolute value of Log2 (Fold change) >1.0 and adj. P value<0.05 were set as cut-offs in identifying DEGs.

Gene ontology (GO) analysis is an approach to identifying associations between genes and biological processes (BP), molecular functions (MF), or cellular components (CC) (21). The Kyoto Encyclopedia of Genes and Genomes (KEGG) is a database for storing information about genomes, biological pathways, diseases, and drugs (22). GO annotation and KEGG pathway enrichment analysis of significant DEGs were performed using the clusterProfiler package of the R program (23); a false discovery rate (FDR)<0.05 was considered statistically significant.

We performed Gene Set Enrichment Analysis (GSEA) to investigate differences in biological processes between different subgroups based on the TCGA-BRCA gene expression profiling dataset. The gene set “c2.KEGG.v7.2.symbols.gmt” was downloaded from the MSigDB database (https://www.gsea-msigdb.org/gsea/msigdb/index.jsp) for GSEA. FDR< 0.25 was taken as sufficient for inclusion. The “c2.cp.kegg.v7.2.symbols.gmt” and “c5.bp.v7.2.symbols.gmt” gene sets obtained using Gene Set Variation Analysis(GSVA) (24) and single-sample Gene Set Enrichment Analysis (ssGSEA) methods, respectively, were used to calculate the scores of the relevant pathways based on the gene expression matrix of each sample; the results were displayed using heat maps.

### 2.5 Drug sensitivity analysis

The Genomics of Drug Sensitivity in Cancer (GDSC) database (www.cancerrxgene.org/) is used to identify oncodrug response data and sensitivity markers in the genome (25). We used the pRRophetic algorithm (26) to construct a ridge regression model based on cell line and TCGA-BRCA gene expression profiling to predict the sensitivity of high- vs. low-ERScore groups to common anti-cancer drugs based on IC50 values.

We used the Tumor Immune Dysfunction and Exclusion (TIDE) score, a computational approach based on gene expression patterns, to predict possible tumor treatment responses in immune-checkpoint blockade(ICB) (27). We evaluated associations between high- and low-ERScore groups and tumor immunotherapy indicators, including *TIDE*, *CD8*, and *CD274*, based on the findings of the TIDE analysis.

### 2.6 Immune-infiltration analysis

CIBERSORTx is an approach based on the principle of linear support vector regression to deconvolute the transcriptome expression matrix to estimate the composition and abundance of immune cells in a cell mixture (28). We uploaded the gene expression matrix data (as TPM) to CIBERSORTx (https://cibersortx.stanford.edu) and filtered output samples with p< 0.05 to obtain the immune cell infiltration matrix. Histograms were plotted using the ggplot2 package of the R program to display the distribution of 22 immune cell infiltrates in each sample. The stromal, immune, and ESTIMATE scores were also calculated based on mRNA expression profiles using the ESTIMATE package of the R program (29).

### 2.7 Construction of an ERScore-based clinical prediction model

To obtain an individualized assessment of patient prognosis using ERScore combined with clinicopathological features, we analyzed the relationship between ERScore, age, and staging, constructing a clinical prediction model using multivariate Cox regression analysis. ERScore, combined with patient age and stage, was selected for inclusion in the model, and a clinical prediction nomogram was constructed. Harrell’s consistency index was determined to quantify discrimination performance. Calibration curves were generated by comparing predicted values of the nomogram with actual survival to assess the performance of the nomogram and the accuracy of the timeROC assessment model.

### 2.8 Cell lines and Western-blot analysis

Normal mammary epithelial cell line MCF-10A and human breast cancer cell lines (MDA-MB-231, SKBR-3, T-47D) were obtained from American Type Culture Collection (ATCC). MDA-MB-231 and T-47D cells were cultured in Dulbecco’s modified Eagle’s medium (DMEM; Hyclone, USA) supplemented with 10% fetal bovine serum (FBS; Gibco, USA) and 1% penicillin-streptomycin (Gibco, USA). SKBR-3 cells were maintained in RPMI-1640 medium (Gibco, USA), supplemented with 10% FBS and 1% penicillin-streptomycin. Mammary Epithelial Basal medium (MEBM; Lonza, Switzerland) was used to culture MCF-10A cells. Cells were incubated at 37 °C with 5% CO2.

For Western-blot analysis, the total proteins of all cells were harvested and lysed with RIPA lysis buffer and separated on SDS-PAGE, then transferred onto nitrocellulose membranes (Millipore, USA). The membranes were blocked with 5% skim milk and incubated with primary antibodies for FBXO6(1:1000, 67697-1-Ig, Proteintech, China), PMAIP1 (1:1000, BM5042, Boster, China), ERP27 (1:1000, ab181172, Abcam, USA), CHAC1(1:1000, 15207-1-AP, Proteintech, China), or β-actin (1:2000, 3700, CST, USA) at 4°C overnight and secondary antibodies for Anti-rabbit IgG (1:4000, 7074, CST, USA) or Anti-mouse IgG(1:4000, 7076, CST, USA) at room temperature for 1 h. The bands were visualized using a Tanon 5500.

### 2.9 Statistical analysis

All data processing and analysis were performed using R software (version 3.6.2). Continuous variables with a normal distribution were analyzed using an independent Student t-test, while non-normally distributed categorical variables were analyzed using the Mann–Whitney U test (the Wilcoxon rank sum test). The chi-square or Fisher exact test was used to compare and analyze the significance between the two groups of categorical variables. The survivor package in R was used to perform survival analysis. Kaplan–Meier survival curves were plotted to show differences in survival, and the log-rank test was used to identify significant differences in survival time between the two groups of patients. All statistical p values were two-sided, and statistical significance was set at p< 0.05.

## 3 Results

### 3.1 Screening for ERS-gene-independent prognostic factors and identification of ERScore

**Figure 1:**
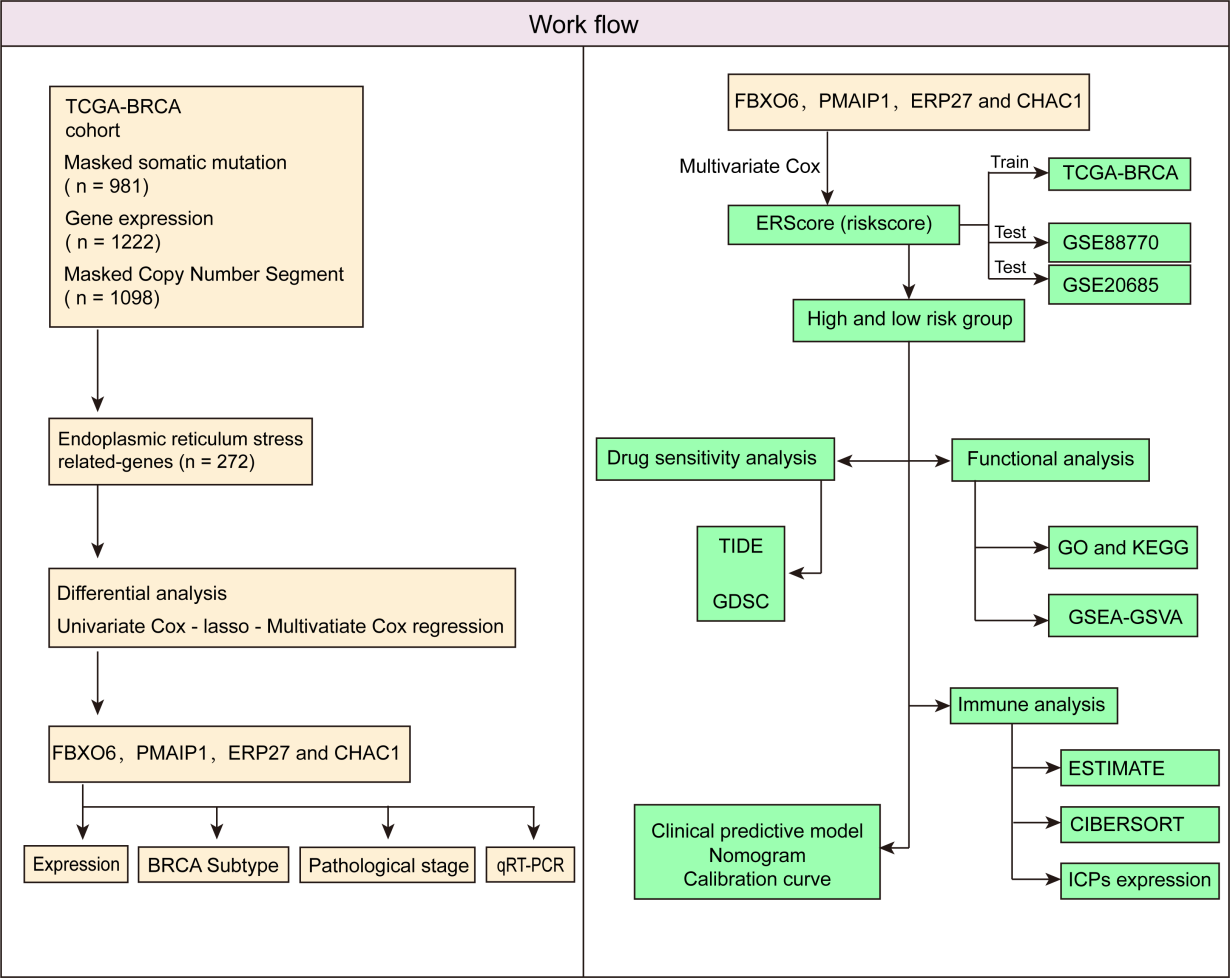
Schematic workflow of the study.

**Figure 2:**
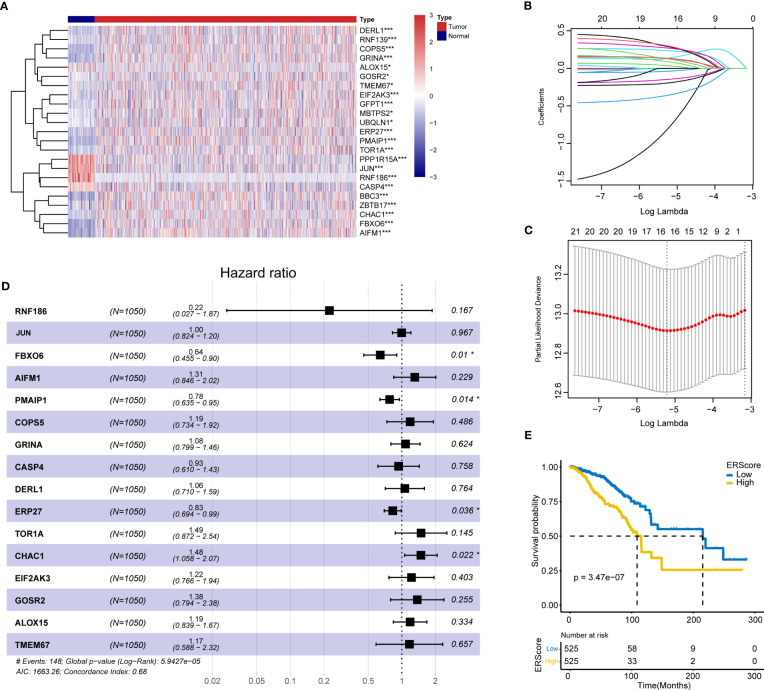
Screening of ERS genes for independent prognostic factors and identification of ERScore. **(A)** Heat map of differentially expressed endoplasmic reticulum (ER) genes. **(B)** The variation curve of the coefficients of the variables with λ penalty. **(C)** Parameter plot of the penalty term with log(λ) values in the lower horizontal coordinates and degrees of freedom in the vertical coordinate. The model bias value was minimized when the variable was 16. **(D)** Multivariate Cox regression forest plot with *FBXO6*, *PMAIP1*, *ERP27*, and *CHAC1* as independent prognostic factors. **(E)** Classification of patients with breast cancer into high- and low-ERScore groups based on median ERScore values; patients in the high-ERScore group had a worse prognosis. *p<0.05, ***p<0.001.

The schematic workflow of our study is shown in **Figure 1**. We identified 23 DEGs, among which 9 and 14 genes had low and high expression in the tumor tissue (**Figure 2A**). We applied LASSO analysis for dimensionality reduction, introducing a penalty factor, λ, and observed that the model was most accurate when the number of variables (genes) was 16 (**Figures 2B, C**).

Multivariate Cox analysis(stepwise, method = “both”)revealed that only *FBXO6*, *PMAIP1*, *ERP27*, and *CHAC1* were independent prognostic factors (**Figure 2D**). Based on the expression levels and the coefficient of variation obtained from the multivariate Cox analysis, we calculated the risk score for this model and defined this as ERScore. Using the median value of ERScore, BRCA patients were divided into high- and low-scoring groups. We observed that patients with a higher ERScore had a significantly worse prognosis (**Figure 2E**).

### 3.2 Key ERS-related gene expression profiles and clinical relevance

**Figure 3:**
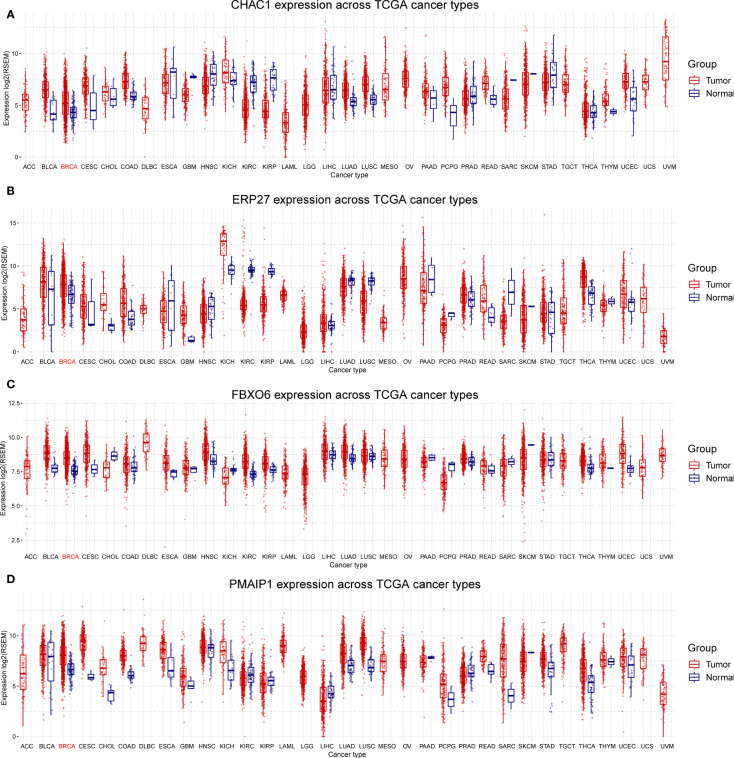
Differential expression of *FBXO6*, *PMAIP1*, *ERP27*, and *CHAC1* in breast and other cancer types. Expression of *CHAC1* **(A)**, *ERP27* **(B)**, *FBXO6* **(C)**, and *PMAIP1* **(D)** was elevated in breast cancer samples but results were inconsistent for other cancer types.

We analyzed the expression of the four independent prognostic factors (*FBXO6*, *PMAIP1*, *ERP27*, and *CHAC1*) in BRCA and 32 other cancer types and observed that all four genes were highly expressed in BRCA. However, we also noted large variations in gene expression between different tumors. (**Figures 3A–D**). We analyzed cancer stage- and BRCA-subtype-specific gene expression. The expression profiles for *FBXO6*, *PMAIP1*, *ERP27*, and *CHAC1* varied substantially (**Figure S1**). Specifically, we observed that *CHAC1* expression was significantly lower in patients with early stage cancer and increased as the malignancy advanced. Additionally, *CHAC1* expression varied significantly between different BRCA subtypes, with the highest expression in the basal types (**Figures S1A, B**). *ERP27* expression showed an opposite trend, as demonstrated by an increase in stages of later cancer (**Figure S1C**), and varied significantly between various BRCA subtypes (**Figures S1E, F**). In contrast, *PMAIP1* expression decreased with advances in the cancer stage, and expression was highest in the LumA subtype (**Figures S1G, H**).

### 3.3 Distribution and validation of ERScores

**Figure 4:**
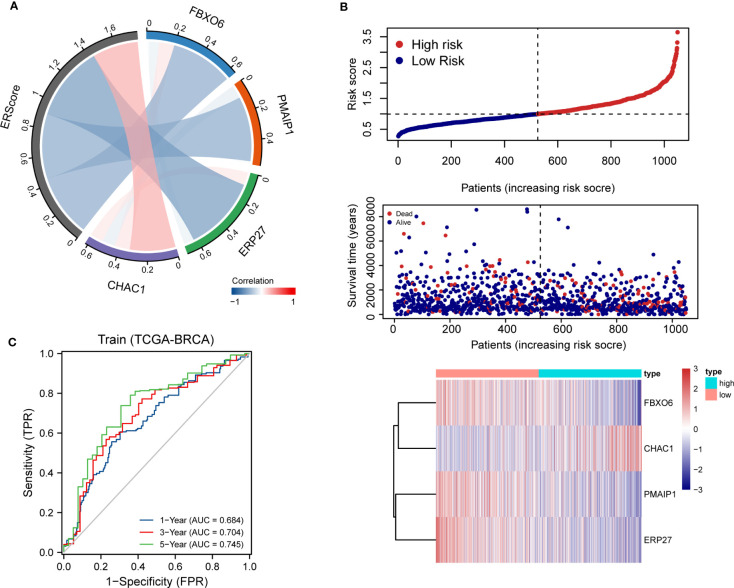
Distribution and validation of ER scores. **(A)** Correlation analysis of ERScore with *FBXO6*, *PMAIP1*, *ERP27*, and *CHAC1*, positive correlation with *CHAC1*, and negative correlation with the remaining genes. **(B)** A risk factor heat map of ERScore displaying the relationship between ERScore and patient survival, as well as the abundance of *FBXO6*, *PMAIP1*, *ERP27*, and *CHAC1* in the ERScore group. **(C)** Time-dependent receiver operating characteristic curves of ERScore for the Cancer Genome Atlas-Breast Invasive Carcinoma (TCGA-BRCA) dataset.

Using penalty coefficients for the four key genes, gene expression levels were multiplied by the corresponding coefficients and summed to create scores, with a final score being calculated for each sample. Based on the scores and gene expression values for patients, we generated a chord diagram and heat maps for risk factors (**Figures 4A, B**). Additionally, time-dependent ROC curve analysis of the scores indicated that the scores had good predictive power for OS in BRCA patients. Notably, the AUC was 0.684, 0.704, and 0.745 for one-, three-, and five-year OS, respectively (**Figure 4C**). We selected the BRCA datasets GSE20685 and GSE88770 for external data testing. We evaluated the model after normalizing the data and observed that the GSE20685 time-dependent ROC curve exhibited an AUC value of 0.722, 0.653, and 0.654 for one-, three-, and five-year OS, respectively. In GSE88770, we selectively analyzed time-dependent ROC at three, four, and five years, corresponding to AUCs of 0.720, 0.863, and 0.779, respectively, owing to a lack of early mortality events. The results indicate that the ERS model has good generalizability (**Figures S2A, B**).

### 3.4 ERScore-based differential gene identification and functional enrichment

**Figure 5:**
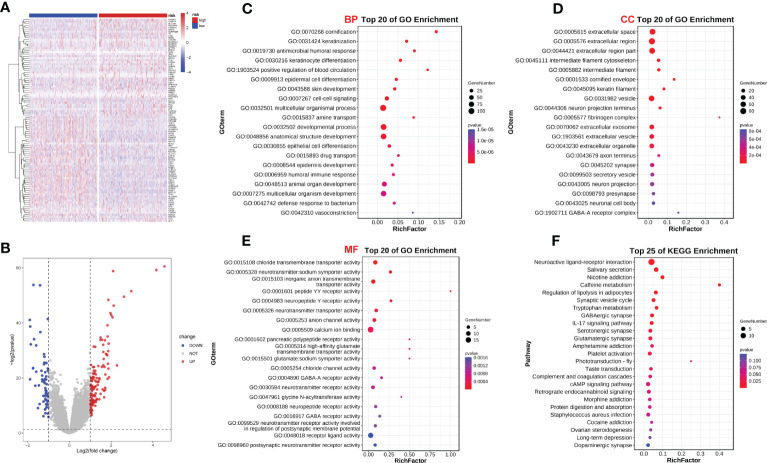
ERScore-based identification of differentially expressed genes (DEGs) and functional enrichment. **(A)** The volcano plot depicting DEGs for patients with breast cancer represented in the TCGA-BRCA dataset and having high or low-ERScore; **(B)** Volcano map displaying similarities in expression; **(C)** Gene Ontology (GO) analysis indicated that the DEGs are associated with biological processes including GO:0070268 cornification, GO:0031424 keratinization, and GO:0019730 antimicrobial humoral response. **(D)** GO analysis revealed that DEGs are associated with molecular functions including GO:0015108 chloride transmembrane transporter activity and GO:0005328 neurotransmitter: sodium symporter activity. **(E)** GO analysis demonstrated that DEGs are associated with cellular components including GO:0005615 extracellular space and GO:0005576 extracellular region. **(F)** Kyoto Encyclopedia of Genes and Genomes (KEGG) analysis revealed that DEGs are involved in pathways including ko04080 neuroactive ligand-receptor interaction, ko04970 salivary secretion, and ko05033 nicotine addiction.

We analyzed the effect of ERScore on the progression of breast cancer by dividing patients in TCGA-BRCA dataset into high- and low-ERScore groups based on median expression values. We identified 197 significant DEGs in the BRCA patients, of which 135 were significantly upregulated and 62 were significantly downregulated (**Figures 5A, B**). We performed functional enrichment analysis on these 197 DEGs. GO analysis revealed significant enrichment of DEGs associated with the biological processes of GO:0070268 cornification, GO:0031424 keratinization, and GO:0019730 antimicrobial humoral processes; molecular functions including GO:0015108 chloride transmembrane transporter activity and GO:0005328 neurotransmitter: sodium symporter activity; and cellular components including GO:0005615 extracellular space and GO:0005576 extracellular region (**Figures 5C–E**). KEGG analysis suggested that significant DEGs were mainly associated with ko04080 neuroactive ligand-receptor interaction, ko04970 salivary secretion, and ko05033 nicotine addiction pathways (**Figure 5F**). Detailed results of the GO and KEGG analyses are presented in **Tables 2**, **3**.

**Table 2:** Top 50 GO analysis enrichment results.

ID	Description	P value	Q value
GO:0070268	cornification	1.53E-14	4.83E-11
GO:0005615	extracellular space	1.40E-11	5.40E-09
GO:0005576	extracellular region	6.94E-11	1.34E-08
GO:0044421	extracellular region part	1.21E-10	1.55E-08
GO:0031424	keratinization	7.40E-10	1.17E-06
GO:0019730	antimicrobial humoral response	1.71E-09	1.79E-06
GO:0030216	keratinocyte differentiation	8.27E-09	6.52E-06
GO:1903524	positive regulation of blood circulation	4.36E-08	2.75E-05
GO:0045111	intermediate filament cytoskeleton	3.53E-07	3.41E-05
GO:0009913	epidermal cell differentiation	1.34E-07	7.05E-05
GO:0043588	skin development	1.99E-07	8.95E-05
GO:0007267	cell-cell signaling	3.01E-07	1.18E-04
GO:0032501	multicellular organismal process	3.78E-07	1.32E-04
GO:0005882	intermediate filament	2.07E-06	1.60E-04
GO:0015837	amine transport	7.15E-07	2.25E-04
GO:0001533	cornified envelope	4.63E-06	2.98E-04
GO:0032502	developmental process	1.17E-06	3.35E-04
GO:0045095	keratin filament	6.09E-06	3.36E-04
GO:0031982	vesicle	7.31E-06	3.53E-04
GO:0048856	anatomical structure development	1.99E-06	5.23E-04
GO:0030855	epithelial cell differentiation	2.79E-06	6.75E-04
GO:0044306	neuron projection terminus	1.59E-05	6.80E-04
GO:0015893	drug transport	3.96E-06	8.91E-04
GO:0008544	epidermis development	4.65E-06	9.78E-04
GO:0006959	humoral immune response	5.62E-06	1.11E-03
GO:0048513	animal organ development	6.39E-06	1.18E-03
GO:0007275	multicellular organism development	6.80E-06	1.19E-03
GO:0042742	defense response to bacterium	7.53E-06	1.25E-03
GO:0005577	fibrinogen complex	5.24E-05	2.02E-03
GO:0042310	vasoconstriction	1.46E-05	2.19E-03
GO:0097756	negative regulation of blood vessel diameter	1.46E-05	2.19E-03
GO:0051047	positive regulation of secretion	1.58E-05	2.27E-03
GO:0046903	secretion	1.71E-05	2.32E-03
GO:0043152	induction of bacterial agglutination	1.81E-05	2.32E-03
GO:0045907	positive regulation of vasoconstriction	1.89E-05	2.32E-03
GO:0051046	regulation of secretion	1.92E-05	2.32E-03
GO:0007610	behavior	2.07E-05	2.41E-03
GO:0070062	extracellular exosome	9.61E-05	3.37E-03
GO:0015108	chloride transmembrane transporter activity	6.77E-06	3.38E-03
GO:0005328	neurotransmitter: sodium symporter activity	1.26E-05	3.38E-03
GO:1903561	extracellular vesicle	0.0001187	3.59E-03
GO:0043230	extracellular organelle	0.0001211	3.59E-03
GO:0019229	regulation of vasoconstriction	3.49E-05	3.93E-03
GO:0051952	regulation of amine transport	3.86E-05	4.20E-03
GO:0010817	regulation of hormone levels	4.04E-05	4.23E-03
GO:0035296	regulation of tube diameter	4.52E-05	4.23E-03
GO:0050880	regulation of blood vessel size	4.52E-05	4.23E-03
GO:0097746	regulation of blood vessel diameter	4.52E-05	4.23E-03
GO:0048731	system development	4.57E-05	4.23E-03
GO:0035150	regulation of tube size	4.77E-05	4.30E-03

**Table 3:** KEGG enrichment pathway analysis results.

Class	ID	Description	P value
Environmental Information Processing	ko04080	Neuroactive ligand-receptor interaction	2.08E-06
Organismal Systems	ko04970	Salivary secretion	1.68E-04
Human Diseases	ko05033	Nicotine addiction	4.57E-04
Metabolism	ko00232	Caffeine metabolism	7.69E-04
Organismal Systems	ko04923	Regulation of lipolysis in adipocytes	2.32E-03
Organismal Systems	ko04721	Synaptic vesicle cycle	5.26E-03
Metabolism	ko00380	Tryptophan metabolism	7.35E-03
Organismal Systems	ko04727	GABAergic synapse	8.97E-03
Organismal Systems	ko04657	IL-17 signaling pathway	1.08E-02
Organismal Systems	ko04726	Serotonergic synapse	1.96E-02
Organismal Systems	ko04724	Glutamatergic synapse	2.13E-02
Human Diseases	ko05031	Amphetamine addiction	2.42E-02
Organismal Systems	ko04611	Platelet activation	2.84E-02
Organismal Systems	ko04745	Phototransduction - fly	3.51E-02
Organismal Systems	ko04742	Taste transduction	3.53E-02
Organismal Systems	ko04610	Complement and coagulation cascades	4.72E-02
Environmental Information Processing	ko04024	cAMP signaling pathway	4.81E-02
Organismal Systems	ko04723	Retrograde endocannabinoid signaling	4.90E-02

**Figure 6:**
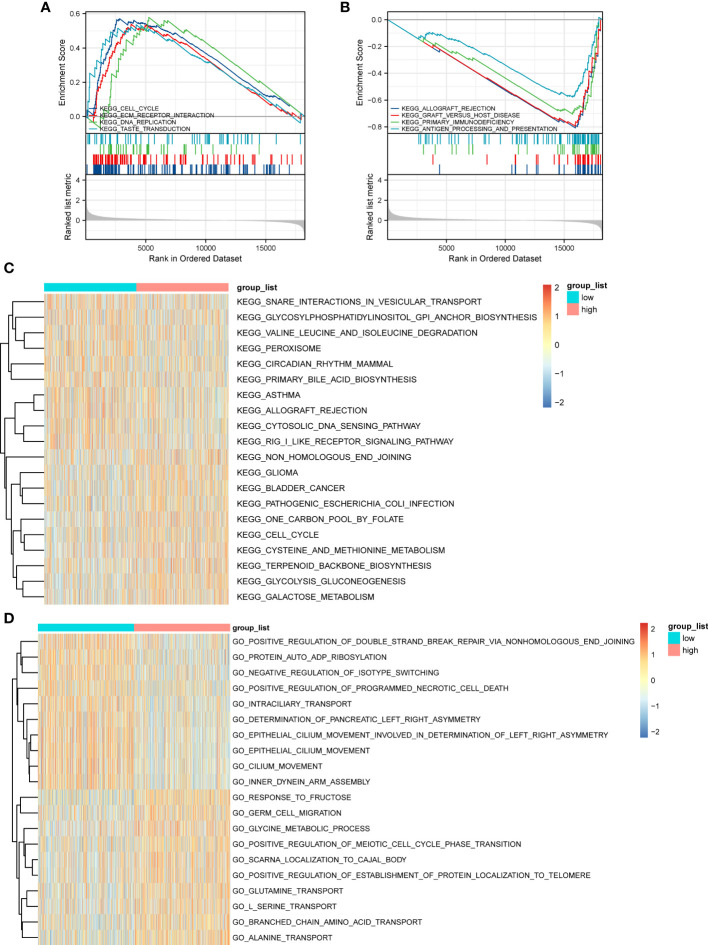
Gene Set Enrichment Analysis (GSEA) and GSVA outcomes. **(A)** KEGG cell cycle, ECM-receptor interaction, and DNA replication genes are significantly enriched in the high-ERScore group. **(B)** KEGG allograft rejection, graft-versus-host disease, and primary immunodeficiency genes are significantly enriched in the low-ERScore group. **(C)** GO positive regulation of double-strand break repair *via* nonhomologous end-joining and protein auto-adduct ribosylation genes are elevated in the high-ERScore group. **(D)** GO alanine and branched-chain amino acid transport genes are elevated in the low-ERScore group.

GSEA based on the c2.KEGG set revealed that cell cycle, ECM-receptor interaction, and DNA replication were significantly enriched in the high-ERScore group. Conversely, allograft rejection, graft-versus-host disease, and primary immunodeficiency were significantly enriched in the low-ERScore group (**Figures 6A, B**). Detailed GSEA results for metabolism-related pathways are presented in **Table 4**. Results from GSVA-KEGG were consistent with those from GSEA analysis. Furthermore, GO analysis indicated that ERScore was associated with multiple biological functions, including positive regulation of double-strand break repair *via* nonhomologous end-joining and protein auto-ADP-ribosylation. Notably, we observed that these functions were negatively correlated with ERScore, suggesting that high-ERScore may be associated with impaired DNA repair and dysregulated protein modification (**Figures 6C, D**).

**Table 4:** GSEA enrichment analysis results.

ID	setSize	NES	FDR
KEGG_CELL_CYCLE	124	1.89	6.75E-02
KEGG_ECM_RECEPTOR_INTERACTION	84	1.70	6.75E-02
KEGG_DNA_REPLICATION	36	1.60	1.63E-01
KEGG_BLADDER_CANCER	41	1.60	1.49E-01
KEGG_TASTE_TRANSDUCTION	51	1.57	1.34E-01
KEGG_GLIOMA	65	1.42	2.51E-01
KEGG_AXON_GUIDANCE	128	1.40	2.06E-01
KEGG_CALCIUM_SIGNALING_PATHWAY	177	1.36	1.78E-01
KEGG_NEUROACTIVE_LIGAND_RECEPTOR_INTERACTION	271	1.34	1.45E-01
KEGG_FOCAL_ADHESION	197	1.32	2.44E-01
KEGG_PATHWAYS_IN_CANCER	322	1.31	1.60E-01
KEGG_CELL_ADHESION_MOLECULES_CAMS	128	-1.32	2.18E-01
KEGG_CYTOKINE_CYTOKINE_RECEPTOR_INTERACTION	261	-1.44	1.51E-01
KEGG_JAK_STAT_SIGNALING_PATHWAY	155	-1.55	1.09E-01
KEGG_NATURAL_KILLER_CELL_MEDIATED_CYTOTOXICITY	131	-1.55	1.47E-01
KEGG_DRUG_METABOLISM_OTHER_ENZYMES	51	-1.57	1.43E-01
KEGG_BETA_ALANINE_METABOLISM	22	-1.58	2.25E-01
KEGG_LEISHMANIA_INFECTION	69	-1.62	1.21E-01
KEGG_VIRAL_MYOCARDITIS	68	-1.69	8.83E-02
KEGG_T_CELL_RECEPTOR_SIGNALING_PATHWAY	107	-1.77	9.18E-02
KEGG_CYTOSOLIC_DNA_SENSING_PATHWAY	53	-1.84	8.78E-02
KEGG_HEMATOPOIETIC_CELL_LINEAGE	85	-1.92	9.18E-02
KEGG_AUTOIMMUNE_THYROID_DISEASE	50	-1.93	8.83E-02
KEGG_INTESTINAL_IMMUNE_NETWORK_FOR_IGA_PRODUCTION	46	-2.12	8.83E-02
KEGG_ANTIGEN_PROCESSING_AND_PRESENTATION	80	-2.18	8.87E-02
KEGG_ASTHMA	28	-2.20	8.20E-02
KEGG_TYPE_I_DIABETES_MELLITUS	41	-2.25	8.44E-02
KEGG_PRIMARY_IMMUNODEFICIENCY	35	-2.31	8.44E-02
KEGG_GRAFT_VERSUS_HOST_DISEASE	37	-2.65	8.44E-02
KEGG_ALLOGRAFT_REJECTION	35	-2.65	8.44E-02

### 3.5 Correlation of ERScore with mutation characteristics

**Figure 7:**
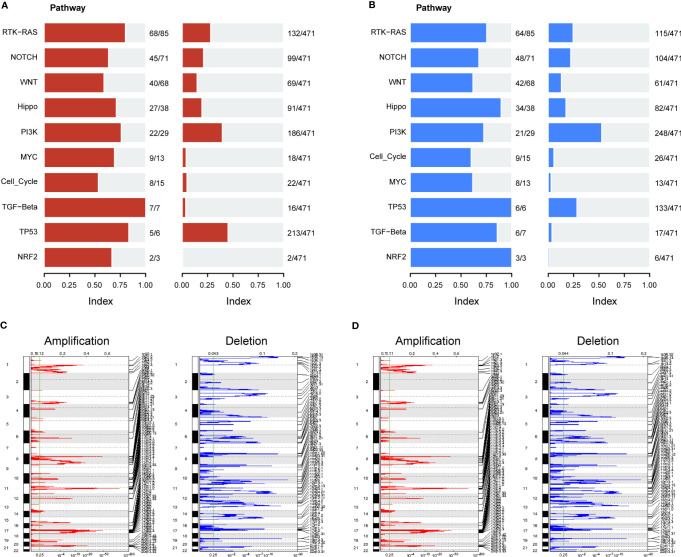
Correlation between ERScore and mutational characteristics. **(A)** Oncopathway enrichment pathways in patients in the high-ERScore group. **(B)** Oncopathway enrichment pathways in patients in the low-ERScore group. **(C)** Copy number amplification and deletion distribution in patients in the high-ERScore group; amplification and deletion are depicted in red and blue, respectively. **(D)** Copy number amplification and deletion distribution in patients in the low-ERScore group; amplification and deletion are depicted in red and blue, respectively.

We further evaluated the association of ERScore with alterations in the expression of genetic variants in BRCA patients. Using the maftools package, we analyzed oncogenic pathway alterations (Oncopathway) associated with the high- and low-ERScore groups but observed no significant differences between the two groups (**Figures 7A, B**). CNV did not significantly differ between the high- and low-ERScore groups (**Figures 7C, D**). Additionally, we calculated various gene set scores reflecting tumor mutation characteristics, including DNA replication and damage repair, using GSVA; no significant differences were observed in the high- and low-ERScore groups (**Figure S3**). The results suggest that ERScore is not significantly associated with genetic variant alterations in tumors and that the role of the underlying changes is more likely to be evident at the transcriptional or post-transcriptional levels.

### 3.6 Association of ERScore with drug sensitivity

**Figure 8:**
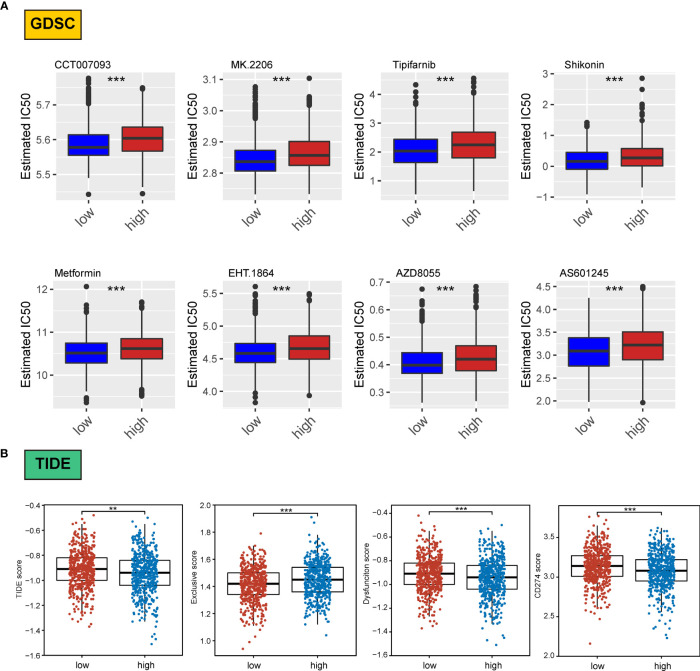
Association of ERScore with drug sensitivity. **(A)** The top eight drugs with differences between the high- and low-ERScore groups based on the Genomics of Drug Sensitivity in Cancer database, ranked by p value. All reflect higher sensitivity for patients in the low-ERScore group. **(B)** Tumor Immune Dysfunction and Exclusion (TIDE), immune escape, immune dysfunction, and CD274 scores based on TIDE calculations. The results suggest that the high-ERScore group is less responsive to immunotherapy. **p<0.01, ***p<0.001.

We assessed differences in the sensitivity phenotypes of common antineoplastic drugs by high- and low-scoring groups through the GDSC database. The test results revealed that 43 of the 138 drugs assessed significantly differed between the two groups. Box plots revealed that patients in the low-scoring group were more sensitive to eight drugs (**Figure 8A**).

Owing to the pivotal role of immunotherapy in treating tumors, we assessed the sensitivity of patients in the high- and low-scoring groups to immunotherapy using the TIDE algorithm. TIDE scores were higher in the high-scoring group than in the low-scoring group, suggesting that the immunotherapy responsiveness was worse in the high-scoring group than in the low-scoring group. Furthermore, the immune escape and immune deficiency scores, as well as the CD274 score, suggested that patients in the high-scoring group were more likely to be less responsive to immunotherapy (**Figure 8B**).

We assessed differences in sensitivity phenotypes for common antineoplastic drugs between high- and low-ERScore groups using the GDSC database. The test results revealed that 43 of the 138 drugs assessed showed significant differences between the two groups. Box plots revealed that patients in the low-ERScore group were more sensitive to 8 drugs (**Figure 8A**).

Owing to the pivotal role of immunotherapy in treating tumors, we assessed the sensitivity of patients in the high- and low-ERScore groups to immunotherapy using the TIDE algorithm. TIDE scores were higher in the high-ERScore group than in the low-ERScore group, suggesting that the immunotherapy responsiveness was lower in the high-ERScore group. The immune escape and immune deficiency scores, along with the CD274 score, suggested that patients in the high-ERScore group were more likely to have a poor response to immunotherapy (**Figure 8B**).

### 3.7 ERScore and immune infiltration

**Figure 9:**
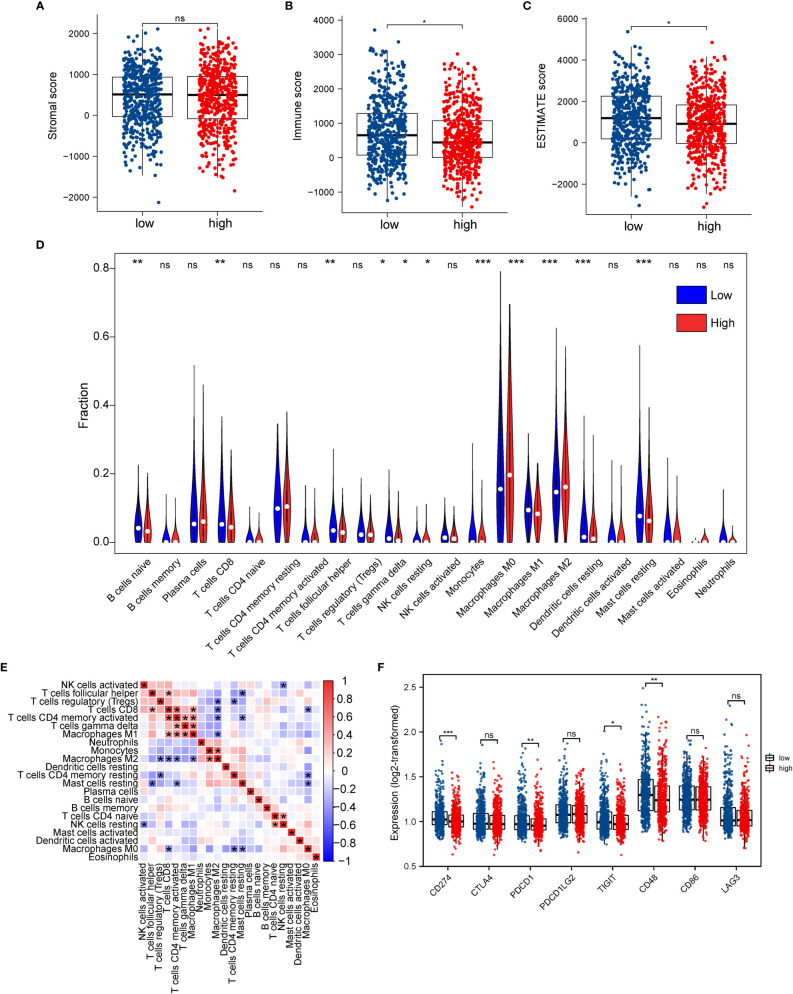
Association between ERScore and immune infiltration. **(A–C)** Stromal cell infiltration, immune cell infiltration, and Estimation of STromal and Immune cells in MAlignant Tumor tissues using Expression data (ESTIMATE) scores in the tumor microenvironment obtained using the ESTIMATE algorithm. Stromal cell numbers do not significantly differ between the two groups, immune cell infiltration is reduced in the high-ERScore group, and tumor purity is higher in the low-ERScore group. **(D)** Differences in immune cell infiltration in the high- and low-ERScore groups. **(E)** Correlation analysis among 22 immune cells. **(F)** Differences in expression of common immune checkpoint genes in the high- and low-ERScore groups. *p<0.05, **p<0.01, ***p<0.001; ns is not significant.

We assessed immune and stromal cell infiltration in the TME using the ESTIMATE algorithm. Our findings were that stromal cell infiltration did not differ significantly between the high- and low-ERScore groups (**Figure 9A**). However, immune cell infiltration was significantly elevated in patients in the low-ERScore group (**Figure 9B**). The ESTIMATE score demonstrated a consistent trend of immune cell infiltration (**Figure 9C**). These results, together with the previous TIDE results, suggest that elevated immune cell infiltration in the low-ERScore group suggests that tumors in this group are closer to a “hot tumor” state and might be more responsive to immunotherapy.

We evaluated the association of ERScore with the overall immune profile and levels of immune cell infiltration in patients represented in TCGA-BRCA dataset. We observed that the levels of multiple immune infiltrating cells differed between the high- and low-ERScore groups. The changes included enhanced infiltration of M0 macrophages and M2 macrophages in the high-ERScore group, whereas cells such as CD8 T cells and mast cells were more abundant in the low-ERScore group (**Figure 9D**). Correlation analysis among immune cells indicated that macrophages, monocytes, B cells, and CD4 and CD8 cells, which are responsible for antigen presentation, did not show a significant correlation. Despite significantly different correlations among multiple immune cells, complex interplay mechanisms existed among these (**Figure 9E**). We thus extracted common immune checkpoint genes, including PD-1 (*PDCD1*), PD-L1 (*CD274*), and *CTLA4*, and assessed differences between high- and low-ERScore groups. The results indicated that the expression of *CD274*, *PDCD1*, and other immune checkpoint genes was elevated in the low-ERScore group, suggesting that the low-ERScore group may have a better immunotherapy response (**Figure 9F**).

### 3.8 Construction of an ERScore-based clinical prognostic model

**Figure 10:**
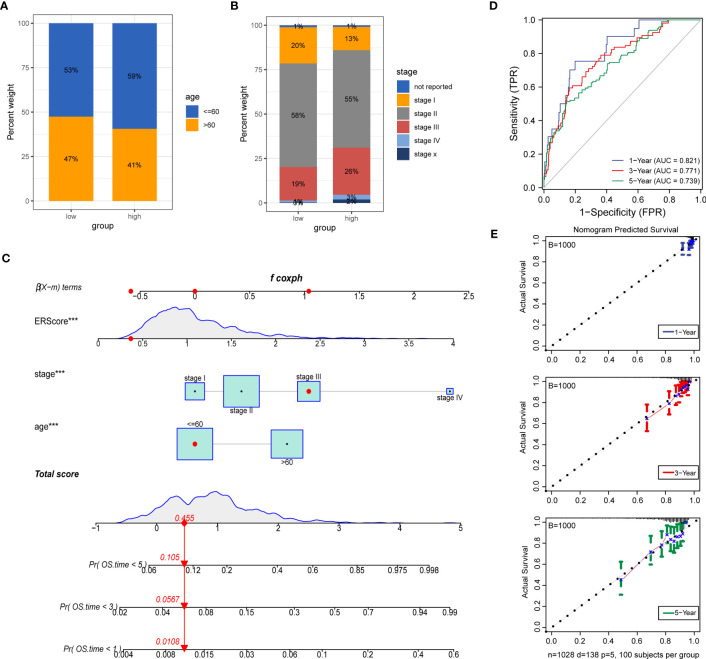
Construction of a clinical prognostic model based on ERScore. **(A, B)** Superimposed histogram depicting age and stage proportions among patients in the high- and low-ERScore groups. Age proportions are similar for patients in both groups, with significantly more patients at an early stage in the low-ERScore than in the high-ERScore group. **(C)** Construction of nomogram, a clinical prediction model based on cuproptosis-related risk scores combined with clinicopathological features, where the red arrow indicated the simulated score for the first patient in the dataset. **(D)** ROC curve of the model over time, with one-, three-, and five-year area under the curve values of 0.821, 0.771, and 0.738, respectively; **(E)** The calibration curve of the nomogram, using the bootstrap method with 1,000 resamplings; the x- and y-axes represent survival obtained from the prediction of the column graph and the actual observed survival with 1,000 repetitions, respectively; the curve indicates that the model has good predictive value for overall survival prognosis in patients at one, three, and five years. ***p<0.001.

To further explore the potential value of ERScore for clinical purposes, we analyzed age and TNM stages for patients in the high- and low-ERScore groups. The results revealed that the age and sex of patients in the two groups were slightly different, with a decreased proportion of older men in the high-ERScore group. The proportion of Stage I and II patients in the low-ERScore group was higher than that in the high-ERScore group, indicating that early stage patients constituted a majority in the low-ERScore group (**Figures 10A, B**).

We constructed prognostic models based on ERScore and clinicopathological factors (age and TNM stage) for patients with breast cancer and visualized these using a nomogram (**Figure 10C**). We validated the accuracy of the model using time-dependent ROC curves and noted highly accurate AUCs of 0.821, 0.771, and 0.739 at one, three, and five years (**Figure 10D**). Furthermore, we used calibration curves to assess the consistency of the model and found good agreement between the OS estimates of the model at one, three, and five years and actual observations with patients (**Figure 10E**).

### 3.9 Validation of four ERS-associated genes in breast cancer cells

**Figure 11:**
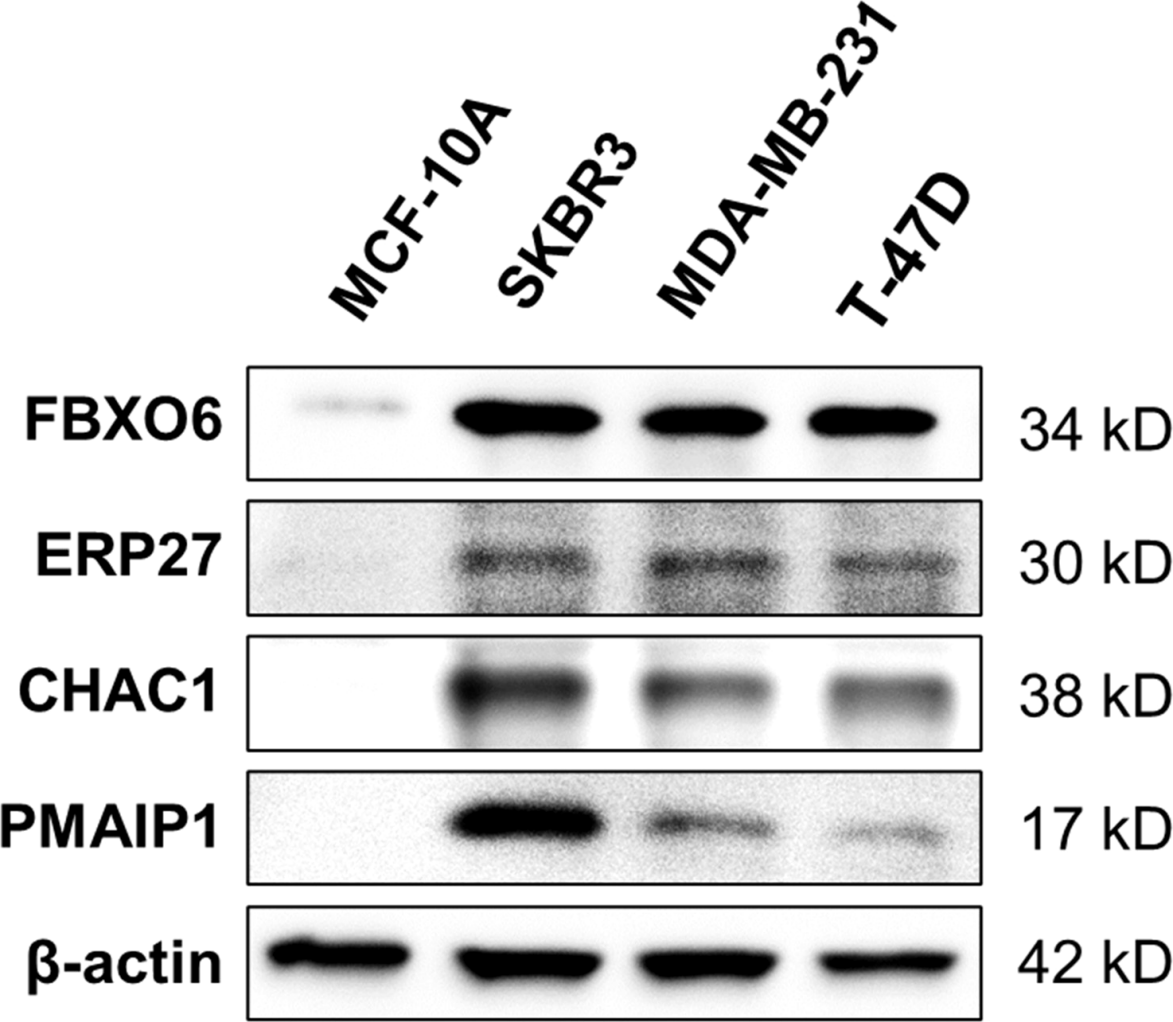
Expression of four endoplasmic-reticulum-stress-related genes. Representative Western-blots show the protein expression of FBXO6, PMAIP1, ERP27, and CHAC1 in different cell lines. β-actin served as the normalization control.

Western-blot analysis revealed that the protein expression of *FBXO6*, *PMAIP1*, *ERP27*, and *CHAC1*were significantly higher in breast cancer cell lines(SKBR-3, MDA-MB-231, T-47D)than in normal mammary epithelial cell line MCF-10A (**Figure 11**).

## 4 Discussion

Growing evidence suggests that current pathological indicators (e.g., estrogen receptor (ER), progesterone receptor(PR), human epidermal growth factor receptor 2(HER2), Ki67, and grading) have limitations for predicting breast cancer prognosis (30). Novel models are thus needed to predict prognosis, enhance personalized treatment, and identify early diagnostic and therapeutic prognostic targets and criteria for patients with breast cancer. We compared the expression profiles of 272 ERS-related genes in primary breast tumors and normal breast tissue and identified *FBXO6*, *PMAIP1*, *ERP27*, and *CHAC1* as independent prognostic factors with established risk models (defining the risk scores as ERScore) and model validation. Collectively, our results suggest that the ERS model has robust and stable predictive prognostic ability by which drug sensitivity, immune infiltration, and prognostic outcome for patients with breast cancer can be accurately predicted.

Additionally, we observed that the four identified genes were significantly more highly expressed in breast cancer samples and differed significantly among tumor subtypes. For example, *FBXO6* expression did not significantly differ with breast cancer stage, while *PMAIP1* expression was lower in late stages. Notably, *ERP27* expression exhibited a decreasing trend with stage, while that of *CHAC1* was low in patients at early stages and increased at later stages. *PMAIP1* (NOXA) reportedly induces apoptosis as a BCL-53 family pro-apoptotic factor in triple-negative breast cancer (31). This is consistent with our observation that *PMAIP1* expression was lower at later stages. Similarly, the overall high *CHAC1* expression in breast cancer samples significantly impacted patient prognosis and survival. Therefore, high *CHAC1* expression in breast cancer may be a vital indicator for diagnostic and prognostic analysis (32). Similarly, we observed that *CHAC1* expression was low in patients at early stages and increased during the later stages. Collectively, these results suggest that *CHAC1* acts as a tumor promoter (33), and *PMAIP1* (34) and *ERP27* act as tumor suppressors in breast cancer. However, a role for *ERP27* has not been reported; therefore, we aim to further examine the underlying molecular mechanisms through *ex vivo* experiments.

In this study, a prognostic model was constructed based on ERS-related scores and clinicopathological factors, including age and TNM stage, for patients with breast cancer to better predict prognosis. Our study suggested that patients in the high-ERScore group had a significantly worse prognosis (p = 3.47e-07), which is consistent with previous ERS-related basic studies (35). Furthermore, our data suggest that patients with a high-ERScore are more resistant to common antitumor agents, and immune cell infiltration is significantly lower in patients with a high ERScore, implying that the tumors in these patients are closer to a “cold tumor” state. Finally, We used Western-blot analysis to detect the protein expressions of four genes in breast cancer cell lines and obtained results consistent with our prediction. However, our study has some limitations. First, the datasets we used to construct and validate the ERS-related prognostic model were obtained from TCGA and GEO. ERscore does not reflect well the prediction of multiple breast cancer subtypes as the there were insufficient cases available in the datasets. Hence, further exploration with clinical samples shall be conducted in due course of time. Second, we only performed preliminary studies and model building for four genes related to ERS. No further functional analysis and mechanistic studies were performed to validate specific biological functions or identify exact signaling pathways. Nonetheless, we were able to successfully construct a prognostic risk model for ERS in breast cancer and validate the reliability and sensitivity, thereby providing a novel viable and reliable predictive tool that may benefit patients with breast cancer.

## Data availability statement

The original contributions presented in the study are included in the article/**Supplementary Material**. Further inquiries can be directed to the corresponding authors.

## Author contributions

Concept and design: RL, SC, JZ and PF. Drafting of the manuscript: PF, JW, and RlL. Critical revision of the manuscript: JW, KC, RlL, LL, and YW. Obtained funding: RlL and JZ. Administrative, technical, or material support: KC, CJ, ZW and BZ. Supervision: RL, SC and JZ. All authors contributed to the article and approved the submitted version.
