# Supplementary material for: Development and validation of an endoplasmic reticulum stress-related molecular prognostic model for breast cancer
Source: Front Oncol. 2023 May 29;13:1178595. doi: 10.3389/fonc.2023.1178595 (PMC10258344; doi:10.3389/fonc.2023.1178595)
Supplement: Supplementary file 1 [file DataSheet_1.docx]

Supplementary Material

Development and validation of an endoplasmic reticulum stress-related molecular prognostic model for breast cancer

Pengyu Fan†, Jiajia Wang†, Ruolei Li†, Kexin Chang, Liuyin Liu, Yaping Wang, Zhe Wang, Bo Zhang, Cheng Ji, Jian Zhang*, Suning Chen*, Rui Ling*

*** Correspondence:** Prof. Rui Ling(lingrui0105@163.com), Dr. Suning Chen ([chsning@fmmu.edu.cn](mailto:chsning@fmmu.edu.cn)) and Prof. Jian Zhang([biozhangj@fmmu.edu.cn](mailto:biozhangj@fmmu.edu.cn))

# Supplementary Figures and Table

## Supplementary Figures

**
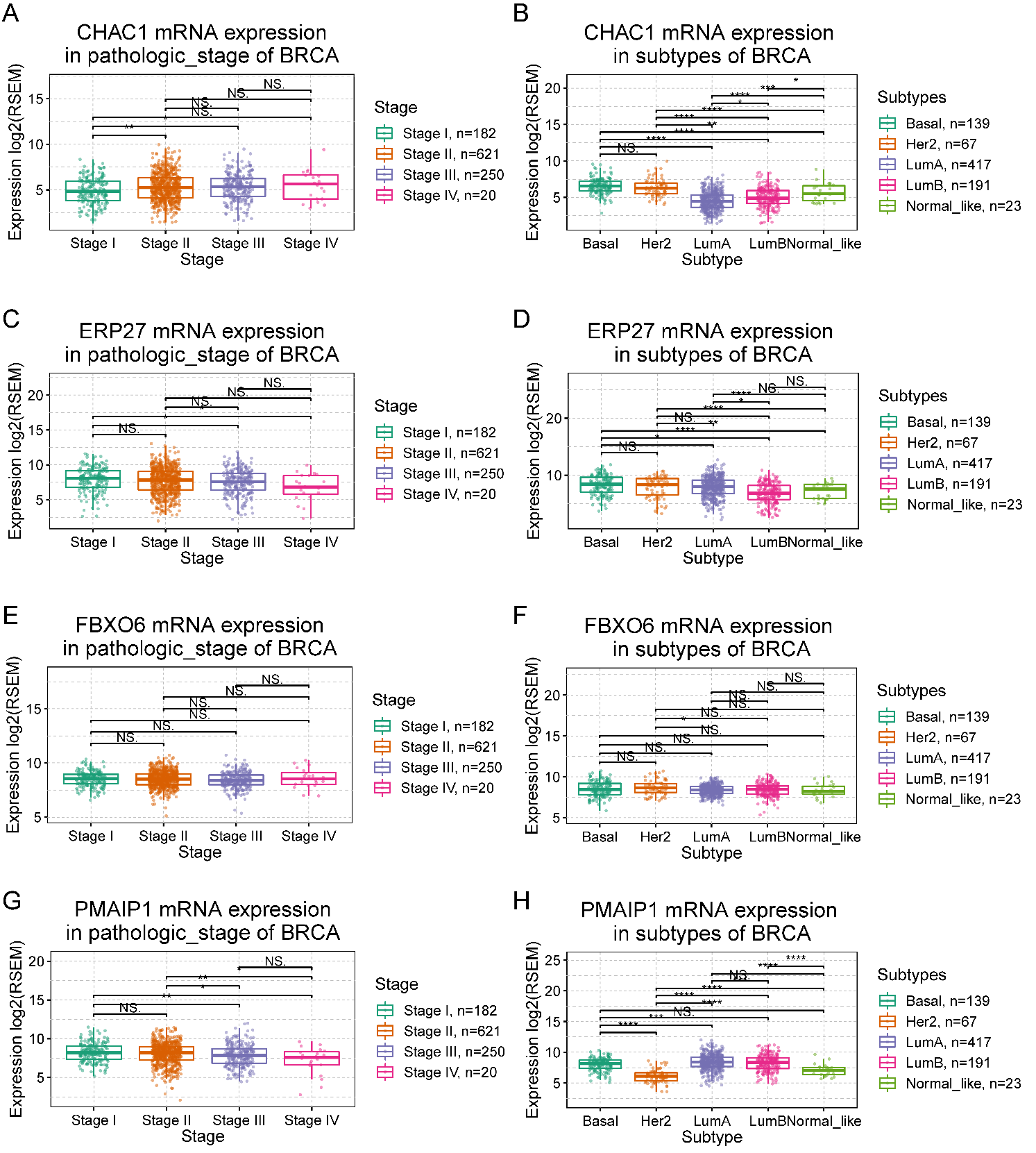
**

**Figure S1.** **Expression differences of *FBXO6*, *PMAIP1, ERP27* and *CHAC1* in breast cancer staging and typing.** (A-B) Differential expression of *CHAC1* in pathological staging and typing. (C-D) Differential expression of *ERP27* in breast cancer staging and typing. (E-F) Differential expression of *FBXO6* in breast cancer staging and typing. (G-H) Differential expression of *PMAIP1* in breast cancer staging and typing.

**
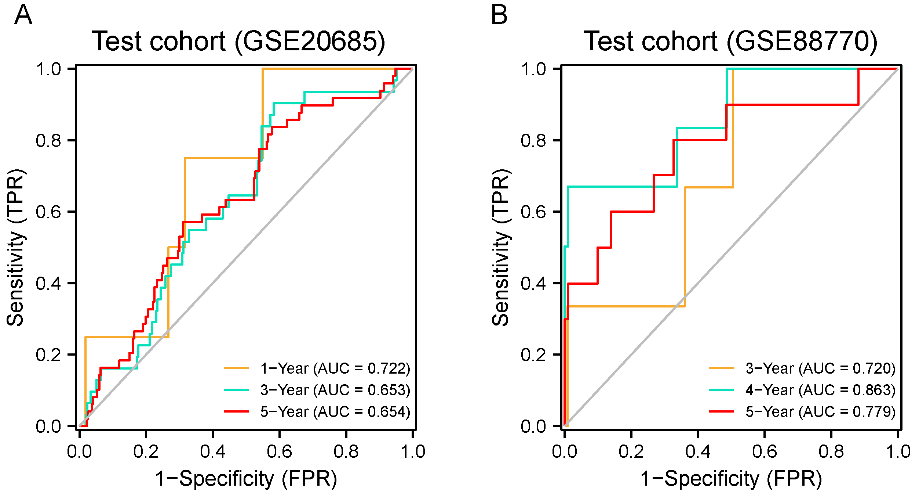
**

**Figure S2. Endoplasmic reticulum stress score from an external dataset test. (**A) ERScore time-dependent ROC curves based on the GSE20685 dataset. The AUCs for 1, 3, and 5 years were 0.722, 0.653, and 0.654, respectively. (B) ERScore time-dependent ROC curves based on the GSE88770 dataset, where the AUCs for 1-, 3- and 5-year were 0.720, 0.863, and 0.779, respectively.

**
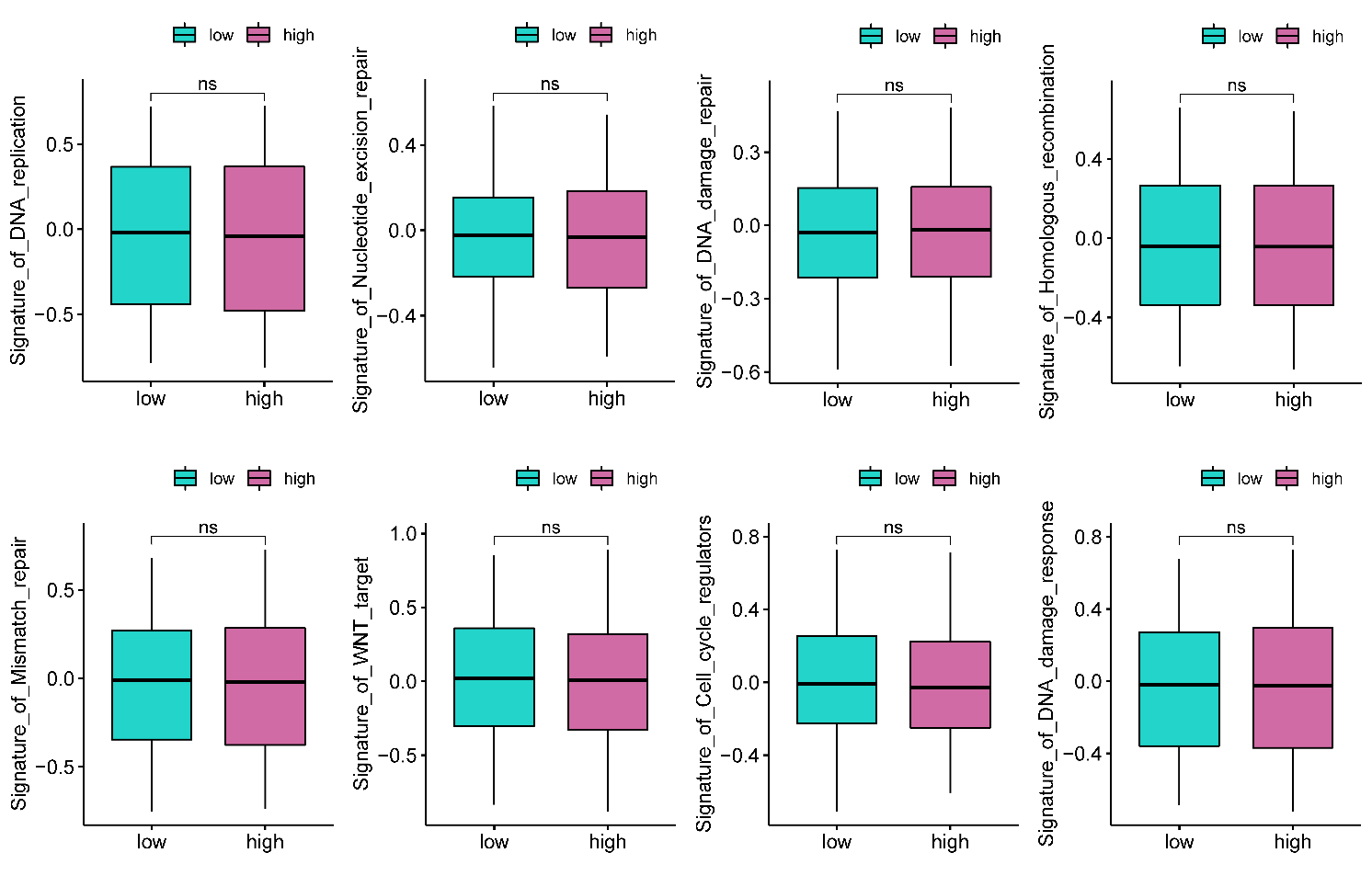
**

**Figure S3. Gene set scoring of tumor mutation feature.**

## Supplementary Table

**Table S1. GEO Dataset Details**

| **Dataset** | **Platform** | **Data type** | **Description** | **Samples (Breast cancer)** |
| --- | --- | --- | --- | --- |
| GSE88770 | GPL570 | Expression profiling by array | [HG-U133_Plus_2] Affymetrix Human Genome U133 Plus 2.0 Array | 117 |
| GSE20685 | GPL570 | Expression profiling by array | [HG-U133_Plus_2] Affymetrix Human Genome U133 Plus 2.0 Array | 327 |
